# Incipient speciation between host-plant strains in the fall armyworm

**DOI:** 10.1186/s12862-022-02008-7

**Published:** 2022-04-27

**Authors:** Karine Durand, Sudeeptha Yainna, Kiwoong Nam

**Affiliations:** 1 DGIMI, Univ. Montpellier, INRAE, Montpellier, France; 2 CIRAD, UPR AIDA, Montpellier, France

**Keywords:** Ecological speciation, Fall armyworm, Genome hitchhiking, Speciation, Speciation with gene flow, *Spodoptera frugiperda*, Sympatric speciation

## Abstract

**Background:**

Recent advancement in speciation biology proposes that genetic differentiation across the whole genome (genomic differentiation, GD) may occur at the beginning of a speciation process and that GD itself may accelerate the rate of speciation. The fall armyworm (FAW, Spodoptera frugiperda) has been used as a model species to study the process of speciation between diverging host-plant strains. We showed in a previous study that GD between the host-plant strains occurred at the beginning of a speciation process based on a population genomics analysis from a population in Mississippi (USA), providing empirical support for the theoretical prediction. In a recent paper, however, panmixia was reported in FAW based on the genomic analysis of 55 individuals collected from Argentina, Brazil, Kenya, Puerto Rico, and the mainland USA. If panmixia is true, the observed differentiation in Mississippi could be at most a phenomenon specific to a geographic population, rather than a status during a speciation process. In this report, we reanalyzed the resequencing data to test the existence of population structure according to host plants using different bioinformatics pipelines.

**Results:**

Principal component analysis, F_ST_ statistics, and ancestry coefficient analysis supported genetic differentiation between strains regardless of the used bioinformatics pipelines. The strain-specific selective sweep was observed from the Z chromosome, implying the presence of strain-specific divergence selection. Z chromosome has a particularly high level of genetic differentiation between strains, while autosomes have low but significant genetic differentiation. Intriguingly, the re-sequencing dataset demonstrates the spread of *Bacillus thuringiensis* resistance mutations from Puerto Rico to the US mainland.

**Conclusions:**

These results show that a pair of host-plant strains in FAW experience genomic differentiation at the beginning of a speciation process, including Z chromosome divergent selection and possibly hitchhiking effect on autosomal sequences.

**Supplementary Information:**

The online version contains supplementary material available at 10.1186/s12862-022-02008-7.

## Background

Speciation process is hampered by gene flow between a pair of diverging taxa in the absence of a geographic reproductive barrier [1]. If only a few loci are targeted by divergent selection, the rest of the genomic loci could be constantly homogenized by gene flow. In this case, two populations will remain only partially differentiated, and the speciation process is not likely to be completed. According to the 'genic view of speciation' [2], the proportion of genetically differentiated sequences is progressively increased by the ongoing divergent selection, and speciation is completed when genomic differentiation (GD) occurs. Here, we define GD as a status in which the vast majority of genomic regions have genetically differentiated sequences between a pair of diverging populations [3]. Since each event of divergent selection causes genetic differentiation at the targeted site and its linked loci, according to the 'genic view of speciation', GD may occur only if the linked loci occupies a whole genome. However, it is unclear if this evolutionary scenario is realistic in natural populations.

Theoretical predictions, however, show that GD may occur at the beginning of a speciation process, rather than at the end, if the diverging effect of divergent selection dominates the homogenizing effect of gene flow. For example, if a locus is targeted by a very strong divergent selection, such that a selection coefficient is higher than migration rate [4] or recombination rate [5], GD is expected to occur because a migration rate can be effectively reduced across the whole genome. In addition, when mild divergent selection targets a very large number of loci, the combined effect of divergent selection can be sufficiently strong to suppress effective migration rate across a whole-genome and GD can be generated [6, 7]. This speciation process was presented as the genome hitchhiking model [8]).

**Fig. 1:**
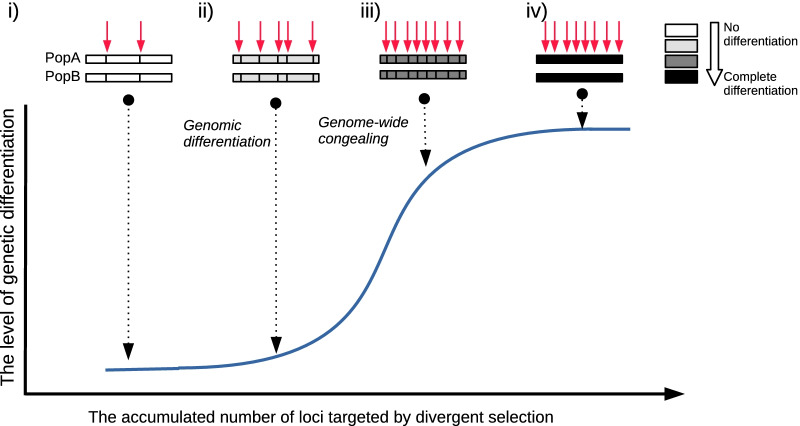
A speciation model involving the genome-hitchhiking[8] and the genome-wide congealing[10]. The X-axis is the number of loci that are targeted by divergent selection, and the y-axis is the level of overall genomic differentiation between two speciating populations (PopA and PopB). (i) Only a few targets are targeted by divergent selection. Selectively targeted loci are differentiated between PopA and PopB while the rest of the genome is undifferentiated by gene flow. (ii) A large number of loci are targeted by mild divergent selection and the combined effect of the mild selection effectively decreases the migration rate between PopA and PopB (the Genome-hitchhiking model). Then, genomic differentiation occurs while the level of differentiation is still low. (iii) The synergistic effect among linkage equilibriums at targets accelerates the rate of genomic differentiation in the presence of following divergent selection (the genome-wide congealing). (iv) Whole-genome sequences are completely differentiated, and the process of speciation is completed

The rate of GD has a non-linear relationship with the accumulated number of loci targeted by divergent selection [4, 9]. Divergent selection creates linkage disequilibrium at the targeted locus. If the number of targeted loci is higher than a certain threshold, the linkage disequilibrium has a synergistic effect between each other and, consequently, the rate of GD is increased. This theoretical prediction was termed genome-wide congealing [10]. According to this theoretical prediction, GD itself may promote the speciation process, instead of being passively generated status (Fig. 1).

The fall armyworm (FAW, *Spodoptera frugiperda*, Lepidoptera: Noctuidae) is native to North and South America, while invasive FAWs populations have been reported from Africa, Asia, and Oceania since 2016 [11]. FAW is observed as two sympatric and morphologically indistinguishable host-plant strains across almost entire native habitat ranges, corn (sfC) and rice strains (sfR) [12]. While more than 353 host plants were reported in FAW [13], these two strains exhibit differentiated ranges of host plants such that sfC prefers corn, sorghum, and cotton, whereas sfR is observed in rice, grasses, and alfalfa [14]. The association between strains and host plants is not absolute, especially because sfR is often found in corn fields. The existence of postzygotic reproductive isolation has been supported by differential fitness of the strains when raised on the original and alternative host plants together with differentiated transcriptional patterns between strains [15]. In addition, hybrids have reduced fertility compared with pure strains [16]. Pre-mating reproductive isolation is also observed from differential mating time and different pheromone blends [17–19]. For this reason, FAWs have been used as a model species to study the speciation process (reviewed in [20]).

In a previous study, we showed that GD between diverging taxa in sympatry may occur at the beginning of the speciation process [3], in line with the genome hitchhiking model. sfC and sfR individuals collected from a single corn field in Mississippi (USA) showed a very low level of genetic differentiation since the genomic average F_ST_ is only 0.0174, while no random grouping of individuals had higher F_ST_ than 0.0174, implying that this level of genetic differentiation cannot be explained by chance. In total, 99.2% of 200 kb windows have genetically differentiated sequences (F_ST_ > 0). We concluded that the combined effect of mild divergent selection may cause GD at the beginning of the speciation process even though this GD does not guarantee the completion of speciation. This GD suggests the condition for genome-wide congealing.

Recently, Schlum et al. reported panmixia among FAW populations through the analysis of 55 samples collected from a wide range of geographic locations including Argentina, Brazil, Kenya, Puerto Rico, and the mainland USA [21], since they did not observe obvious genetic differentiation between sfC and sfR. If genomic differentiation between sfC and sfR is not supported from populations from diverse geographic locations, the observed genomic differentiation from populations in Mississippi [3] should not be considered in the context of speciation because this differentiation might concern only specific geographic populations, rather than a general evolutionary trend in FAW.

We re-used the dataset generated by Schlum et al. to test if population structure according to host plant strains is supported in FAW using different bioinformatics pipelines. First, we used the same methods as Schlum et al. to test if the same trend can be reproduced by performing variant calling for each individual and by merging the resulting files into one. Since they used BBDUK [22], Bwa [23], Bcftools mpileup [24], and ref ver3.1 [25] for read-filtering, mapping, and variant calling, respectively, we denoted this bioinformatics pipeline BBB3-indi. Second, we used slightly different methods by performing variant calling simultaneously across all individuals. This bioinformatics pipeline is BBB3-all here. Third, we used very different bioinformatics pipelines including read-filtering (AdapterRemoval [26]), mapping software (Bowtie2 [23]), variant calling software (GATK HaplotypeCaller [27]), and a reference genome assembly (ver7 [28]). This bioinformatics pipeline was denoted AOG7. Then, we performed population genetics analyses to test whether genetic differentiation between sfC and sfR is supported.

## Results and discussion

The resequencing data contained 42 sfC samples, eight sfR samples, three hybrid samples, and two unknown samples, identified from a single nucleotide position at the TPI exon 4 shown in Additional file 2: Table S1 of Schlum et al. [21]. The numbers of unfiltered SNPs (Single nucleotide polymorphisms) were 96,794,353, 94,191,415, and 78,897,948 for BBB3-indi, BBB3-all, and AOG7, respectively (Table 1). After filtering, the numbers of remaining SNPs were different between BBB3-indi (28,165,218) and BBB3-all (25,263,019) only by 11.49%. However, the number of SNPs from AOG7 was 10,217,767, which was lower than those from BBB3-indi or BBB3-low by 59.56–63.72%. Unexpectedly, the number of SNPs from BBB3-indi was 10.19 times higher than the one in Schlum et al. (2,762,958), even though the same methods were used.

**Table 1:** The number of SNPs generated by different bioinformatics pipelines. The row of Schlum et al. indicates the SNP numbers described in the original paper[21]

Bioinformatics pipeline	Unfiltered SNP	Filtered SNPs
Schlum et al.	120,398,863	2,762,958
BBB3-indi	96,794,353	28,165,218
BBB3-all	94,191,415	25,263,019
AOG7	78,897,948	10,217,767

**Fig. 2:**
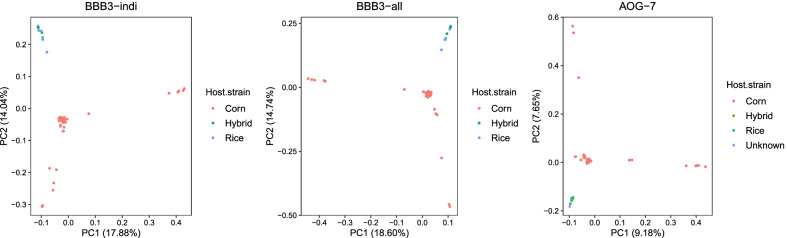
Population structure according to host-plant strains. Principal component analysis was performed from the same raw resequencing data with different bioinformatics pipelines (BBB3-indi, BBB3-all, and AOG-7). Corn and Rice denote sfC and sfR, respectively

We performed principal component analysis (PCA) to test population structure. In all cases of BBB3-indi, BBB3-all, AOG7, sfR was clearly separated from sfC at the second principal component (Fig. 2), implying that the observed pattern is robust against used bioinformatics pipelines. Clustering according to geographic population was not clearly observed (Additional file 1: Fig. S1). This result supports population structure according to strains, even though other evolutionary forces may also play a role in population structure (e.g., the first principle component). The separation between sfC and sfR was not observed from Schlum et al. [21]. Since the same pattern was not reproduced when we used the same raw data and the same bioinformatic pipeline (BBB3-indi), we do not consider the conclusion of Schlum et al. [21] is valid anymore.

We tested genetic differentiation between sfC and sfR. Pairwise Weir and Cockerham’s F_ST_ [29] was 0.067 between sfC and sfR from BBB3-indi. No random grouping out of 200 replications has higher F_ST_ than 0.067, suggesting statistically significant genetic differentiation between sfC and sfR (*p-value* < 0.005). Statistical genetic differentiation between sfC and sfR was also observed from BBB3-all (0.062, *p-value* < 0.005) and AOG7 (0.079, *p-value* < 0.005), again showing that this trend is robust against used methods.

Then, we tested if GD is supported by F_ST_ calculation in 200 kb windows. If > 90% of 200 kb windows have F_ST_ > 0, we considered that the dataset has GD, as we defined in our previous study [3]. We did not use BBB3-indi or BBB3-all, because the used reference genome assembly is highly fragmented (N50 = 52.7 kb) [25] and, consequently, a very large number of 200 kb windows is expected to be truncated. In AOG7, 72.0% of 200 kb windows had F_ST_ > 0. Thus, GD appears not to have occurred yet. This pattern is in contrast with the result from a population from Mississippi, from where 93.8% of 200 kb windows had F_ST_ > 0 [3]. This difference can be explained by differential rates of genomic differentiation among geographic populations or by slightly non-overlapping differentiated regions among geographic populations.

**Fig. 3:**
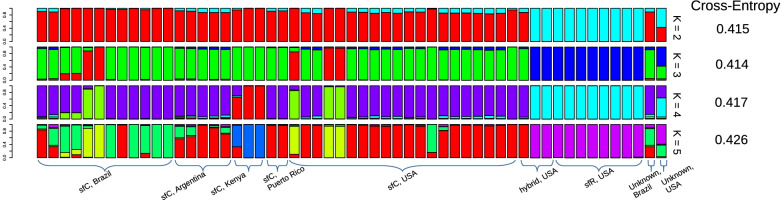
Ancestry coefficient analysis with a range of K-values. Cross-entropy is indicated at right

Ancestry coefficient analysis from AOG7 was performed to infer the genetic relationship among the ancestry of each individual. The ancestry of sfC individuals was diverse while that of sfR individuals had a distinct ancestry from sfC in a wide range of K values (Fig. 3). This result again supports genetic differentiation between sfC and sfR. Intriguingly, both ancestry coefficient analysis and PCA (Additional file 1: Fig. S2) suggested that one and two individuals classified as hybrids could be sfC and sfR, respectively. We speculate the possibility that a single diagnostic nucleotide position at the TPI gene might not be sufficient to classify an individual as a hybrid.

**Fig. 4:**
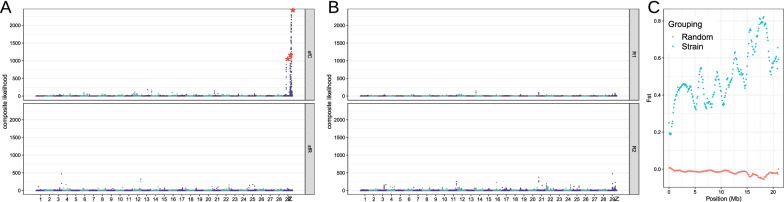
Strain-specific selective sweeps. **A** Composite likelihood of being targeted by selective sweeps at grids along the genome of sfC and sfR. Obvious outliers of composite likelihood are indicated by red asterisks. **B** Composite likelihood of being targeted by selective sweeps calculated from two random groups (R1 and R2). **C** F_ST_ between sfC and sfR and between random groupings calculated from sliding windows along the Z chromosomes. The sizes of windows and steps are 1mb and 100 kb, respectively

If genetic differentiation between strains is promoted by strain-specific divergent selection, selective sweeps are expected to generate strain-specific footprints of selective sweeps as well. Targets of selective sweeps of sfC and sfR were identified from the composite likelihood of being targeted by selective sweep from site frequency spectrums[30]. Three apparent outliers of sfC-specific targets of selective sweeps were observed on the Z chromosome (Fig. 4A). The likelihood in these three peaks ranges between 511.5 and 2299.3, corresponding to the highest 0.140% outliers among total grids. We also calculated the composite likelihood from random grouping, and an apparent outlier like in Fig. 4A was not observed (Fig. 4B). F_ST_ calculated between sfC and sfR exhibited obvious outliers on the Z chromosomes, which was not observed from a random grouping (Fig. 4C). This result supports the hypothesis that genetic differentiation between strains is promoted by strain-specific divergent selection targeting Z-linked genes. This conclusion is in line with a well-known phenomenon that sex chromosomes play disproportionally a greater role in speciation than autosomes, termed either large-X [31] or large-Z effects [32] depending on XY or ZW system.

In total, 139 genes were identified from these three outliers (Additional file 2: Table S1). In total, the function of 93 genes out of 139 genes is unknown. The association between speciation and these genes was unclear. We propose that these genes can be studied further using functional genomics experiments (such as CRISPR/CAS9) to find the function of these genes and their role in a speciation process.

**Fig. 5:**
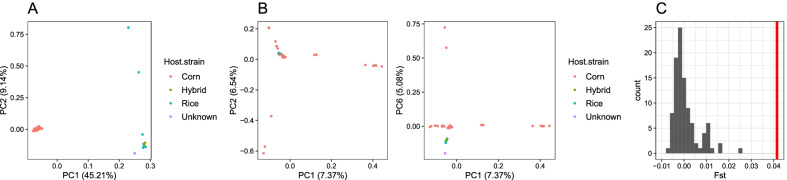
Population structure inferred from Z chromosome and autosomes. Principle component analysis was performed from **A** the Z chromosome and **B** autosomes. **C** The histogram shows the distribution of autosomal F_ST_ calculated between two random groupings with 100 replications. The red horizontal bar indicates autosomal F_ST_ calculated between sfC and sfR

Our observation that selective sweeps were observed only from the Z chromosome suggests a possibility that genetic differentiation between sfC and sfR can be completely explained from the Z chromosome. In this case, population structure according to host plant strains (Fig. 2) might not be observed from autosomal sequences. We performed PCA from the Z chromosome and autosomes separately to test this possibility. Z chromosome exhibited clear grouping according to host plant strains (sfC and sfR) at the first principal component (Fig. 5A). Autosomal PCA results did not show such a population structure at the first or the second principal components (Fig. 5B, left). Grouping according to host plant strains was observed at the sixth principal component (Fig. 5B, right). Autosomal F_ST_ between sfC and sfR was 0.0414, which is far lower than Z chromosome F_ST_ (0.4670). No random grouping out of 100 replications generated higher F_ST_ than 0.0414 on autosomes (Fig. 5C), suggesting significant genetic differentiation between sfC and sfR (p-value < 0.01). This result suggests that the allele frequencies on the Z chromosome were predominantly affected by genetic differentiation between sfC and sfR, this differentiation had a minor effect on the autosomal allele frequencies.

The resequencing data generated by Schlum et al. [21] is very interesting in that this dataset includes information showing that 19 individuals are resistant and three individuals are susceptible to Cry1F, a type of insecticidal *Bacillus thuringiensis* (Bt) toxin. Five causal Cry1F resistance mutations at ABCC2 gene have been reported, including GC bi-nucleotide insertion from a population in Puerto Rico [33, 34] and a 12 bp insertion from another Brazilian population [35], as well as GY deletion, P799K/R substitution, and G1088D substitution in protein sequences from a Brazilian population [36]. We reported in a previous paper that the resistance mutation did not spread to other geographic populations from the originated population [37]. In the resequencing data generated by Schlum et al. [21], two Brazilian individuals have both GY deletion and P799K substitution, implying that this resistant mutation did not spread to geographically remote populations by gene flow. GC insertion was observed from one individual from Puerto Rico and one from North Carolina (USA), supporting that the spread of GC insertion from Puerto Rico to the USA mainland occurred. The G1088D substitution and 12 bp insertion were not observed. Intriguingly, no resistance mutation was observed from 15 resistant individuals. Further analysis is urgently necessary to identify other geographic populations with GC insertions and to identify unknown resistance mutations to make a strategy to control Cry1F resistant FAW.

A possible criticism against genomic differentiation between host-plant strains is that the observed difference between sfC and sfR might be due to genetic differentiation between a population containing the sfR samples and the rest of the populations with different geographic locations. The four individuals out of a total of eight sfR individuals were collected from Puerto Rico, Florida, and Texas, where sfC individuals were also collected. Therefore, the genomic differentiation between sfC and sfR is not likely to represent the differentiation according to geographic populations.

## Conclusion

We showed that FAW experienced genomic differentiation between host-plant strains from the resequencing data of 55 samples collected from a wide range of geographic locations including Argentina, Brazil, Kenya, Puerto Rico, and the mainland USA by Schlum et al. [21], regardless of used bioinformatics pipelines or reference genome assemblies. Z chromosomes have a much higher level of genetic differentiation than autosomes, at least partly due to sfC-specific divergent selection. Autosomal sequences also have weak but significant genetic differentiation between sfC and sfR. Therefore, we propose the possibility that Z chromosome differentiation by divergent selection led to the autosomal differentiation by reducing effective migration rate between sfC and sfR [4]. Since the reported phenotypic differences between sympatric strains have an effect as prezygotic [17–19] or post-zygotic reproductive barriers [15, 16], which are expected to increase genetic differentiation between sfC and sfR, we propose that the observed differentiation should be interpreted in the context of the speciation process.

Since the samples used by Schlum et al. [21] were collected mostly from corn fields, the genetic differentiation between sfC and sfR is not necessarily the consequence of ecological divergent selection on the range of host plants. Instead, the differentiation could be driven by other evolutionary forces, such as differential mating time, different sexual pheromone blends, and intrinsic incompatibility between nuclear and mitochondrial genomes. The exact cause of genetic differentiation could be identified through inbreeding experiments, population genomics analysis, and functional investigation in future studies.

## Methods

The resequencing fastq files generated by Schlum et al. [21] was downloaded from NCBI SRA (SRR12044614-SRR12044668). Then, we treated the raw reads using the same methods described in the original study [21] based on the used scripts. More specifically, adapter sequences and low-quality reads were discarded using BBDuk [22]. Then, the reads were mapped against the ver3.1 reference genome assembly at BioInformatics Platform for Agroecosystem Arthropods (https://bipaa.genouest.org/is/) [25] using Bwa v0.7.17 mem [23], and resulting bam files were generated for each sample. Variant filtering was performed for each bam file using mpileup at bcftools v1.9 [24]. We did not include four samples (SRR12044616, SRR12044617, SRR12044614, and SRR12044618) because they were also excluded in the original paper [21]. The resulting bcf files were merged into one vcf file using vcftools v0.1.15 [38]. If an SNP has a minor allele frequency lower than 0.05 or the proportion of genotyped individuals is less than 50%, the SNP was discarded using vcftools v0.1.15. Lastly, only biallelic SNPs were retained using plink2 [39]. This resequencing data was denoted 'BBB3-indi'.

Next, variant calling was performed from all bam files simultaneously using mpileup at bcftools v1.9 [24]. Then, we discarded SNPs if the minor allele frequency is lower than 0.05 or if the proportion of genotyped individuals is lower than 50%. This resequencing data is denoted 'BBB3-all'.

To generate the third resequencing dataset, adapters and low-quality base pairs were removed from the raw reads using AdapterRemoval v2.1.7 [26]. Reads were mapped against the ver7 reference genome [28] with –very-sensitive-local preset using bowtie2 v2.3.4.1 [40]. Variant calling was performed using GATK v4.1.2.0 HaplotypeCaller [27]. An SNP was discarded if the QD score was lower than 2.0, the FS score was higher than 60.0, the MQ score was lower than 40.0, if the MQRankSum score was lower than -12.5, or the ReadPosRankSum score was lower than -8.0. In addition, if a proportion of genotyped individuals was lower than 80% or if minor allele frequency was lower than 0.01, the SNP was discarded using vcftools v0.1.15 [38]. The resulting resequencing dataset was denoted AOG7.

The information of identified strain was obtained from Additional file 2: Table S1 at Schlum et al. [21]. Here, a single position at TPI exon 4 was used as a marker[41]. Pairwise Weir and Cockerham’s F_ST_ [29] was calculated using VCFtools v0.1.15 [38] for each resequencing dataset. PCA was performed using plink2 [39]. We used sNMF v1.2 [42] for ancestry coefficient analysis. Loci under selective sweep were identified using SweeD v3.2.1 [30]. The number of grids was 1,000 per chromosome.

## Supplementary Information

**Additional file 1: Figure S1.** The result of principal component analysis with the information of sampled locations. **Figure S2.** The principal component analysis shown in Fig. 2 in the main text with+geom_point(position=position_jitter(h=0.1, w=0.1)) function to reduce overlapping among points for visualization purpose. Here, random numbers ranging between 0 and 0.1 were added to both the x-axis and y-axis to each point to spread the points.

**Additional file 2: Table S1.** The list of genes within the identified targets of sfC-specific selective sweeps.

## Data Availability

Computer programming scripts used to generate the SNP data set are available a﻿t GitHub (https://github.com/kiwoong-nam/Speciation_FAW/). The VCF files are available upon request.

## Abbreviations

AOG7: AdapterRemoval, Bowtie2, GATK HaplotypeCaller, and a reference genome of ver7
BBB3-all: BBDUK, Bwa, Bcftools mpileup, and a reference genome of ver3.1, variant calling from all samples
BBB3-indi: BBDUK, Bwa, Bcftools mpileup, and a reference genome of ver3.1, individual variant calling
Bt: *Bacillus thuringiensis*
COX1: Cytochrome c oxidase subunit 1
GD: Genomic differentiation
FAW: Fall Armyworm
PCA: Principal component analysis
sfC: *Spodoptera frugiperda*, Corn strain
sfR: *Spodoptera frugiperda*, Rice strain
SNP: Single nucleotide polymorphism
TPI: Triosephosphate Isomerase
USA: United States of America
