# Supplementary material for: Incipient speciation between host-plant strains in the fall armyworm
Source: BMC Ecol Evol. 2022 Apr 27;22:52. doi: 10.1186/s12862-022-02008-7 (PMC9047287; doi:10.1186/s12862-022-02008-7)
Supplement: Supplementary file 1 — Additional file 1: Figure S1. The result of principal component analysis with the information of sampled locations. Figure S2. The principal component analysis shown in Fig. 2 in the main text with+geom_point(position=position_jitter(h=0.1, w=0.1)) function to reduce overlapping among points for visualization purpose. Here, random numbers ranging between 0 and 0.1 were added to both the x-axis and y-axis to each point to spread the points. [file 12862_2022_2008_MOESM1_ESM.docx]

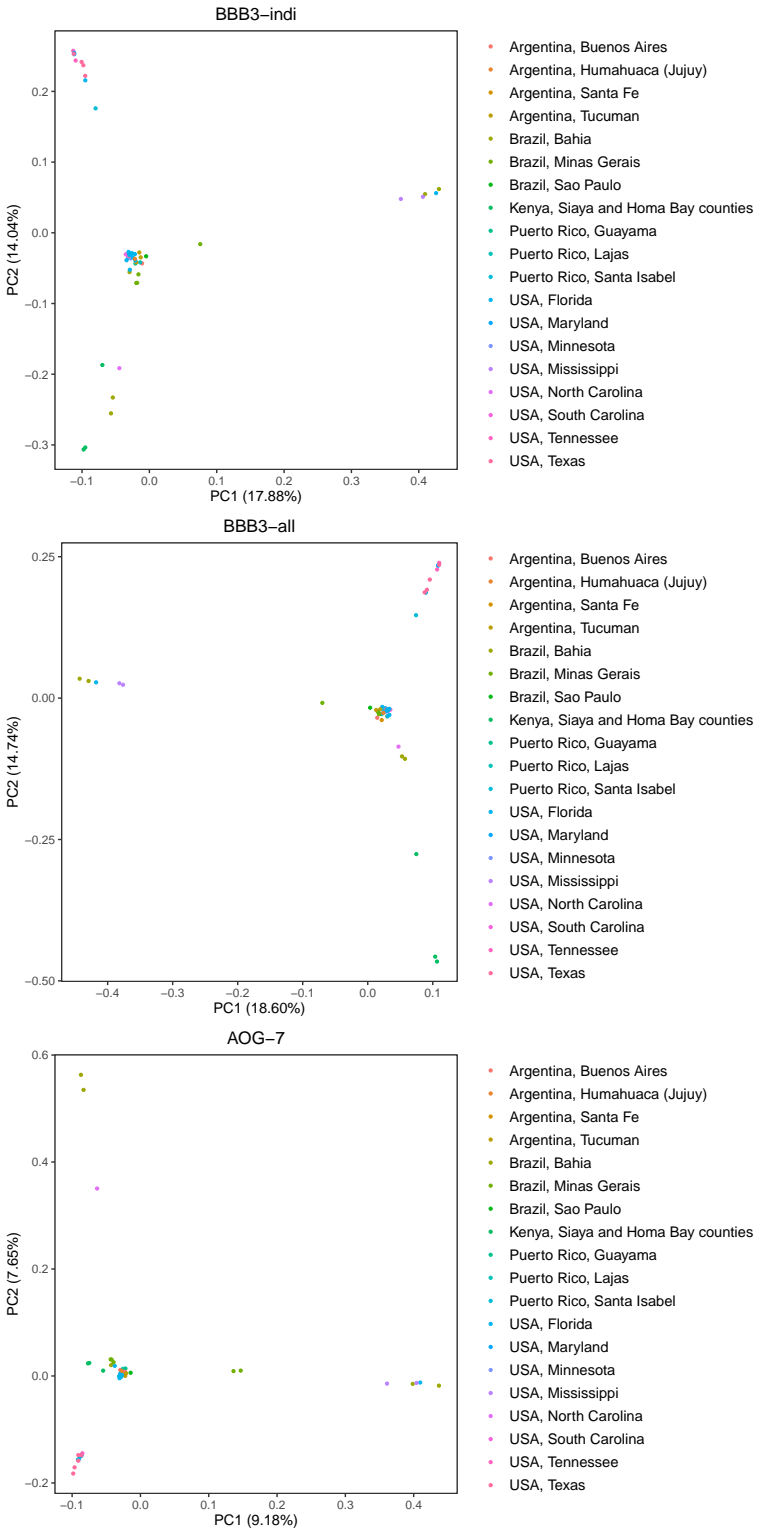
Figure S1. The result of principal component analysis with the information of sampled locations.


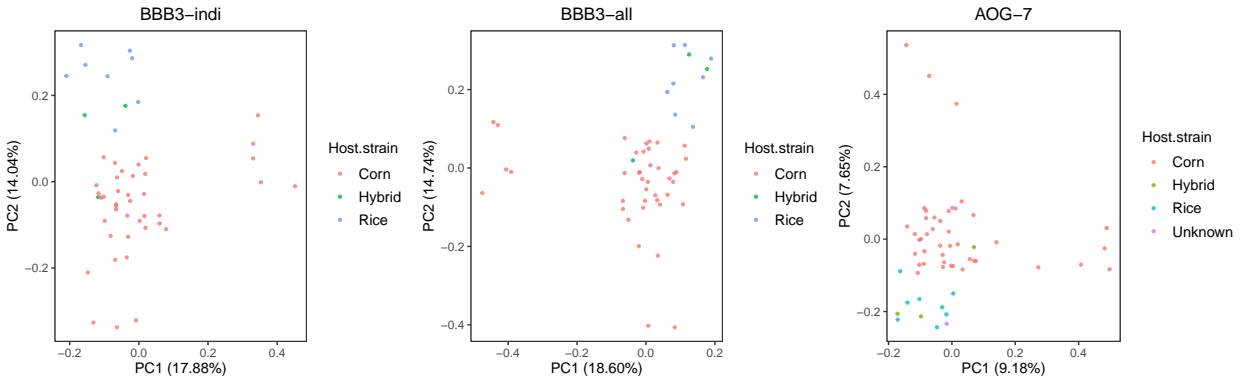
Figure S2. The principal component analysis shown in Figure 2 in the main text with +geom_point(position=position_jitter(h=0.1, w=0.1)) function to reduce overlapping among points for visualization purpose. Here, random numbers ranging between 0 and 0.1 were added to both the x-axis and y-axis to each point to spread the points.
